# Study of Degradation Mechanisms of Strength and Thermal-Physical Properties of Nitride and Carbide Ceramics—Promising Materials for Nuclear Energy

**DOI:** 10.3390/nano12111789

**Published:** 2022-05-24

**Authors:** Askhat Berguzinov, Artem Kozlovskiy, Inesh Kenzhina, Dmitriy I. Shlimas

**Affiliations:** 1 Department of Heat Power Engineering, Toraighyrov University, Pavlodar 140000, Kazakhstan; 2 Laboratory of Solid State Physics, The Institute of Nuclear Physics, Almaty 050032, Kazakhstan; kenzhina@physics.kz (I.K.); shlimas@mail.ru (D.I.S.); 3 Department of General Physics, Satbayev University, Almaty 050032, Kazakhstan; 4 Engineering Profile Laboratory, L.N. Gumilyov Eurasian National University, Nur-Sultan 010008, Kazakhstan; 5 Advanced Electronics Development Laboratory, Kazakh-British Technical University, 59 Tole bi St., Almaty 050000, Kazakhstan

**Keywords:** nitride ceramics, carbides, radiation defects, strength, reactor materials, heavy ions, materials for nuclear power

## Abstract

The dependences of changes in the strength properties of nitride and carbide ceramics under high temperature irradiation with Kr^15+^ and Xe^22+^ heavy ions at irradiation doses of 10^12^–10^15^ ions/cm^2^ are presented in this work. The irradiation was chosen to simulate radiation damage processes that are closest to the real conditions of reactor tests in operating modes of increased temperatures. Polycrystalline ceramics based on AlN, Si_3_N_4_ nitrides, and SiC carbides were chosen as objects of research, as they have great prospects for use as a basis for structural materials for high-temperature nuclear reactors, as well as materials for nuclear waste disposal. During these studies the effect of radiation damage caused by irradiation with different fluences on the change in mechanical strength and hardness were determined, and the mechanisms causing these changes depending on the type of irradiated materials were proposed. The novelty of this study is in the results obtained determining the stability of the strength and thermophysical parameters of nitride and carbide ceramics exposed to high-temperature irradiation, which made it possible to determine the main stages and mechanisms for changing these parameters depending on the accumulated radiation damage. The relevance of this study consists not only in obtaining new data on the properties of structural materials exposed to ionizing radiation, but also in the possibility of determining the mechanisms of radiation damage in ceramics.

## 1. Introduction

One of the promising directions of solving the global energy crisis, as well as solving the problem of hydrocarbons, is the use of alternative energy sources, including nuclear energy [1,2]. The focus of research should be on the new types of nuclear reactors under development [3,4], which have great productivity and safety as compared to the previous generation of nuclear reactors. The interest in these power units is due to the possibility of their operation at higher temperatures, which significantly increases the reactors’ efficiency, as well as to the use of new types of uranium fuel with higher burnup rates. Meanwhile, the increase in the coolant temperature, as well as the core operating temperature, requires the use of new types of reactor materials that have high melting temperatures (above 2000 °C), as well as good indicators of resistance to external influences, including mechanical and radiation damage [5,6,7,8]. There are also additional requirements for these materials used as structural materials, concerning safety and resistance to radiation defects and subsequent effects associated with their accumulation in the damaged layer exposed to irradiation, which can be several tens of microns thick [9,10,11]. The main effects caused by irradiation usually consist in the destruction of crystalline and chemical bonds, as well as disordering, amorphization, or swelling processes [12,13,14,15]. Most of the effects caused are associated with the formation of point defects and dislocations, which occur as a result of the interaction of incident ions with the crystal structure of the material. At the same time, in most cases, point defects are highly mobile, and can also form more complex defect compounds, the presence of which leads to a deterioration in the properties of the material. A change in the dislocation density, in turn, can have a dual effect: in one case (up to a certain amount of accumulated dislocations), a hardening effect can be observed, in the other case, when the dislocation density is too high, destruction and the formation of microcracks can occur. All these effects are the final stages of interaction between radiation and matter, and also have a cumulative effect, leading to negative consequences.

The degree of radiation damage also depends very strongly on the energy of ionizing radiation and the type of particles that interact with the substance. The most destructive consequences, in terms of destruction and amorphization processes, are two types of radiation damage. These are helium or hydrogen embrittlement due to processes associated with transmutation nuclear reactions under the impact of neutrons or during interaction with coolant, and the destruction of the near-surface layer due to interaction with products or fragments of nuclear fuel fission [16,17,18]. In both cases, the most vulnerable layer is the near-surface layer of structural materials, which is directly exposed to radiation. Although the processes of helium or hydrogen embrittlement are quite clear, since the main mechanisms leading to the destruction of the near-surface layer have been identified, there are still quite a few questions about the effects of nuclear fuel fission fragments [19,20].

Firstly, there is no unified theory of defect formation caused by the interaction of heavy charged particles with the structural damage material. Today there are several theoretical and experimental models attempting to describe these processes in various materials that are based on ideas about the energy exchange between the colliding particle and the material structure [18,19,20,21,22].

Secondly, there is no consensus on the nature of this energy exchange for various materials, including dielectrics, for which some of the processes associated with post-radiation relaxation processes are impossible due to the dielectric nature of the material [23,24].

Thirdly, and no less importantly, there are no precise data on the critical doses of radiation damage leading to complete and irretrievable material degradation. This is due to the fact that for new types of structural materials there are still insufficient experimental data on the nature of radiation damage, which opens up great prospects for research [20,21,22,23,24,25].

The most promising materials for new generation reactors are refractory ceramics based on oxides (MgAl_2_O_4_, BeO, Al_2_O_3_, ZrO_2_), carbides (WC, TaC, SiC), and nitrides (AlN, BN, Si_3_N_4_) [20,21,22,23,24,25,26,27,28,29,30]. Interest in them is caused by the possibility of their operation at increased temperatures, as well as in various aggressive environments, as they have high resistance to most types of acids and alkalis. Excellent indices of mechanical strength and wear resistance allow their use as structural materials of the first wall or protective casings for nuclear waste disposal. Among the majority of dielectric ceramics, the three most commonly considered types of ceramics can be highlighted: SiC, AlN, Si_3_N_4_. As mentioned above, the interest in their use as structural materials is due to a combination of their properties, as well as the results of a number of scientific studies reflecting the relationship between the degree of radiation damage and the degradation resistance of these materials. However, in spite of the sufficient amount of experimental data, there are still a number of unresolved questions that require clarification and refinement. For the most part, this is primarily associated with the establishment of critical doses of damage during irradiation with high-energy particles, as well as with the establishment of the relationship between structural changes induced by irradiation and mechanical and strength properties.

## 2. Experimental Part

Polycrystalline ceramics AlN, Si_3_N_4_, and SiC were chosen as objects for research. According to a priori data, the selected ceramic samples are polycrystalline structures consisting of grains the sizes of which vary within 70–90 nm, which provides a dislocation density in the initial samples of ~10^10^ 1/cm^2^. Additionally, X-ray phase and energy dispersive analysis showed the absence of any impurities in the composition of ceramics, which excludes the influence of impurity defects on the radiation resistance of ceramics. The choice of objects for research is due to the prospects of their use as structural materials for high-temperature nuclear reactors. At the same time, the choice of nitride ceramics, in particular AlN and Si_3_N_4_, was due to their different mechanisms of radiation damage and the consequences of exposure to heavy ions; and the choice of SiC due to the need to compare changes in the strength of nitride ceramics with one of the traditional materials used in nuclear power and has high rates of radiation damage resistance. The differences in the consequences of radiation damage during heavy ion irradiation in AlN and Si_3_N_4_ consist in the so-called latent tracks found earlier in Si_3_N_4_—discontinuous damaged areas along the trajectory of ions in the material, as well as amorphization of the damaged near-surface layer at doses above 10^13^ ions/cm^2^.

The studied samples were irradiated with Kr^15+^ and Xe^22+^ heavy ions on the DC-60 heavy ion accelerator. Irradiation fluences were 10^12^–10^15^ ions/cm^2^. Irradiation was conducted at 1000 K in order to simulate radiation damage conditions as close as possible to the real influences. The irradiation fluences were chosen due to the simulation possibility of the effects of both the beginning of isolated damaged region formation along the ion trajectory in the material and their overlap, which contributes to the effect of the formation of disordered regions with altered electronic density, as well as structural damage. Table 1 shows the results of the simulation of the values of energy losses of the incident ions during interaction with electron shells and nuclei for two types of ions and all types of investigated ceramics.

As it is visible from the data presented on the energy losses of incident ions in ceramics, their values are close enough for all the chosen types of ceramics, in view of the proximity of the structural properties of the chosen objects for research, and also the density of materials. Changing the type of irradiation from Kr^15+^ ions to Xe^22+^ ions leads to a two-fold increase in energy losses in the interaction with nuclei and one and a half times the interaction with electron shells, while the maximum depth of penetration of ions in the material is increased by less than 10–12%. The total ion penetration depth for the selected types of ions in the materials is 15–16 μm depending on the type of incident ions. In the case of comparison with neutron exposure (during neutron irradiation), the selected fluences and energies of incident ions correspond to atomic displacements of ~0.001–1 dpa, depending on the irradiation fluence. At the same time, it is also necessary to make the clarification that, in contrast to neutron exposure, which affects a great depth (several tens of microns), irradiation with heavy ions is localized in an area not exceeding 15–16 microns, which corresponds to the near-surface layer, which is most susceptible to external mechanical influences.

The determination of strength properties and the dynamics of their changes as a result of radiation damage accumulation was conducted with the application of the indentation method using standard Vickers pyramid at variable load on the indenter. The choice of indenter loading conditions was carried out in order to determine the deformation and reduction in strength properties, along with hardness at different depths. Based on the change in hardness value data compared to the initial values, the degree of softening and destruction of the near-surface layer was determined.

The thermal conductivity properties of the investigated ceramics, as well as the study of thermal conductivity reduction, were carried out using the standard method of determining the longitudinal heat flux, implemented using the KIT-800 unit.

## 3. Results and Discussion

**Figure 1:**
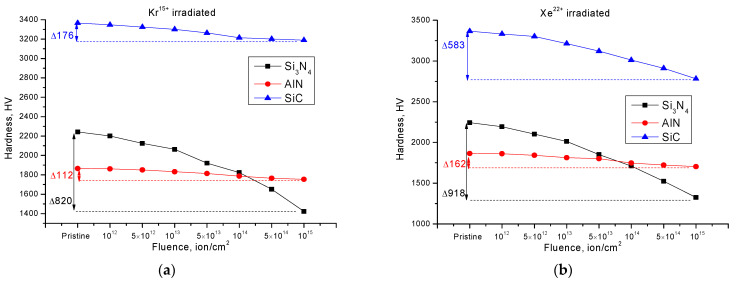
Results of changes in the hardness values of the investigated ceramics depending on the type of incident ions: (**a**) irradiation with Kr^15+^ ions; (**b**) irradiation with Xe^22+^ ions.

Figure 1 shows the results of changes in the hardness value of the surface-damaged layer of ceramics depending on the irradiation fluence and the type of incident ions. The general appearance of changes in hardness values can be characterized by two different stages for all types of ceramics depending on the irradiation fluence. The first stage is characterized by insignificant changes of hardness parameters and softening degree (decrease in hardness parameters) for AlN and SiC ceramics of not more than 0.2–2% for a fluence range of 10^12^–5 × 10^13^ ions/cm^2^. It should be noted that for Si_3_N_4_ ceramics in this range of fluences, the change in hardness values was from 3 to 7–10% depending on the type of incident ions. The second stage of change in strength characteristics is typical for irradiation fluences above 5 × 10^13^ ions/cm^2^ and is characterized by a stronger change in hardness values, as well as an increase in the degree of softening. Such a behavior of the strength properties of ceramics at irradiation fluences of 5 × 10^13^ ion/cm^2^ ions may be due to the fact that with an increase in the irradiation fluence, there is an overlap of defect regions formed during the passage of ions through the material, which at lower fluences remain isolated from each other, which also does not make it possible to increase the probability of the formation of complex defects or dislocation loops. However, for Si_3_N_4_ ceramics the change in strength at these fluences of irradiation is more pronounced than for AlN and SiC ceramics.

It is also worth noting that for Si_3_N_4_ ceramics irradiation with Kr^15+^ and Xe^22+^ ions leads to almost the same decrease in hardness depending on the fluence, and the difference at high irradiation fluences of 10^14^–10^15^ ions/cm^2^ is not more than 3–5%. In the case of AlN ceramics, the hardness degradation at irradiation fluences of 10^14^–10^15^ ions/cm^2^ for both types of ions is approximately the same, while for SiC ceramics, changing the irradiation type leads to a threefold decrease in hardness at high irradiation fluences. These changes of hardness depending on the change in the type of incident ions indicates the different nature of structural changes caused by irradiation in the damaged layer, which leads to different mechanisms of damage and their influence on the resistance of ceramics to degradation.

**Figure 2:**
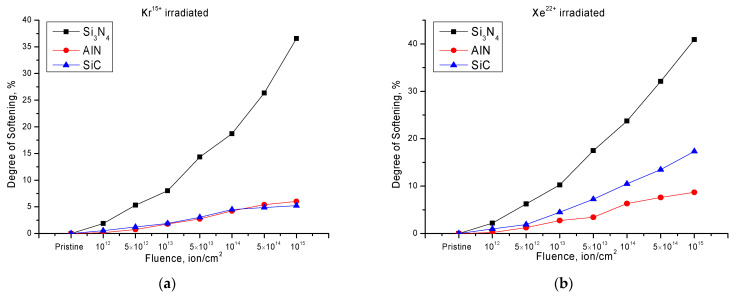
The results of changes in the softening degree of the damaged layer of the investigated ceramics depending on the type of incident ions: (**a**) irradiation with Kr^15+^ ions; (**b**) irradiation with Xe^22+^ ions.

The softening degree of the damaged surface as a result of irradiation was calculated on the basis of the obtained data on the change in hardness values. This value was calculated using Formula (1):

(1) $$Degree\_of\_softening=\frac{H_{0}-H_{irr}}{H_{0}}\times 100\%,$$

where *H*_0_ is the hardness value in the initial state; *H_irr_* is the hardness value in the irradiated state. This value is normalized to 100% in order to show the degradation of strength properties of ceramics depending on the irradiation fluence. The calculation results are presented in Figure 2.

The general type of change in the value of the degree of softening and destruction of the damaged layer of ceramics has a different trend depending not only on the type of irradiated ceramics, but also on the type of incident ions. At the same time, at low irradiation fluences, a small change in the softening degree is observed, which may be due to the fact that the formation of point defects isolated from each other is observed in the structure, which also does not allow them to form complex defects. Thus, for Si_3_N_4_ ceramics the damaged layer destruction has the same tendency for both types of irradiated ions, which at the maximum fluences of irradiation becomes more than 35%, indicating a strong destruction of material and its embrittlement.

For AlN ceramics the change in resistance to softening is much less than for Si_3_N_4_ ceramics and is not more than 5% when irradiated with Kr^15+^ ions and not more than 7–10% when irradiated with Xe^22+^ ions. Meanwhile, at high irradiation fluences of 10^14^–10^15^ ions/cm^2^ for both types of ions, a change in the degradation trend is observed, with its growth decreasing with increasing fluence. Such behavior may be due to the effect of the accumulation of radiation damage and changes in dislocation density, which leads to the effect of radiation hardening and increases resistance to embrittlement and degradation.

For SiC ceramics the change in the degree of softening for different types of ions has a different character. Thus, for samples irradiated with Kr^15+^ ions the change in softening resistance is comparable with the results for AlN ceramics; at high irradiation fluences of 5 × 10^15^–10^15^ ions/cm^2^, SiC ceramics have greater resistance to degradation. However, when irradiated with Xe^22+^ ions, the trend of change in the resistance to softening is close to the results for Si_3_N_4_ ceramics, but the degradation value is much less and at the maximum irradiation fluence was not more than 20%. Such a change in the character of resistance to degradation from irradiation by Xe^22+^ ions in SiC ceramics can be explained by the fact that some of the chemical bonds, Si-Si, Si-C, and C-C, are destroyed due to the large energy losses of the incident ions, thereby creating the additional chemical disorder in the ceramic’s structure whose presence leads to the degradation of the damaged layer. However, at low irradiation fluences, as well as due to high-temperature irradiation, some of the structural deformations in SiC ceramics can be stabilized or annealed by enhancing the dynamic annealing of defects [31].

Let us consider in more detail the processes and mechanisms which can influence changes in the strength properties of ceramics under irradiation. More intensive reductions in the strength characteristics for Si_3_N_4_ ceramics at irradiation fluences higher than 5 × 10^13^ ion/cm^2^ can be caused by the amorphization processes of the damaged near-surface layer. More about the amorphization processes and increases in the degree of structural disorder in Si_3_N_4_ ceramics under irradiation by heavy ions of Xe and Bi was reported in [32,33,34]. The authors, using Raman spectroscopy, showed that the so-called amorphization of the near-surface layer occurs when the fluence exceeds 10^13^ ions/cm^2^ depending on the type of incident ions. Herewith, the authors established visible changes in the near-surface damaged layer throughout the depth of ion penetration into the material. The authors attribute this behavior of the damaged layer, as well as the amorphization of Si_3_N_4_ ceramics, to the effect of overlapping defect regions, the radius of which is estimated to be no more than 2 nm, which at fluences above 10^13^ ions/cm^2^ leads to the appearance of the effect of overlapping defect regions.

In turn, the amorphization processes so evident for Si_3_N_4_ ceramics were not experimentally detected for AlN and SiC ceramics. It is also worth noting that for Si_3_N_4_ ceramics, intermittent latent tracks were found earlier in [32,33,34], the presence of which the authors attribute to the energy losses of the incident ions, as well as the destruction of chemical and crystalline bonds. However, it should be noted that the density of the detected tracks is much less than the irradiation fluence, which indicates that the latent discontinuous tracks are formed by a more complex mechanism in Si_3_N_4_ ceramics than similar structural elements in dielectric polymers. It is also worth noting that the observed effects of the amorphization of the damaged layer are observed throughout the irradiated volume of the material. This fact indicates that the greatest contribution to the processes leading to the destruction and subsequent amorphization is made by electron losses of incident ions, and therefore with a change in the electron density and its anisotropic change along the trajectory of the ions in the irradiated material. In this case, unlike metals, in which the change in the electron density distribution as a result of external influences has a reversible nature, such processes are difficult for dielectric materials. It is also worth noting that for the selected types of ions the electronic losses are approximately equal, but the degradation processes leading to a change in strength characteristics at high fluences of irradiation have a different trend, as well as different degrees of degradation and softening.

It is also worth noting that the investigated samples were irradiated at a temperature of 1000 K, the choice of which is due to the possibility of modeling the effects of radiation damage comparable to the real conditions of operation of materials in high-temperature nuclear reactors. It is well known that during high-temperature irradiation, at low fluences, the effect of the formation of point defects can be leveled by the effect of the thermal annealing of defects associated with changes in the vibrational modes of atoms in the lattice nodes. The change in the thermal vibrational modes of the atoms in the crystal lattice, in some cases, can play a dual role.

On the one hand, as was shown in [35], an increase in irradiation temperature above 773 K leads to a decrease in radiation damage in ceramics, and in the case of irradiation at 1223 K irradiation does not carry visible structural damage, according to X-ray diffraction data. As a result, some of the point defects formed when ions pass through the material can be annealed; this leads to a decrease in the degree of radiation damage in the material. However, this proposal is not quite consistent with the observed effects of strength reduction in Si_3_N_4_ ceramics, for which the strength reduction effect has a fairly pronounced trend throughout the studied range of irradiation fluences. This effect can be explained by the fact that Si_3_N_4_ ceramics are more susceptible to the influence of electronic interactions leading to degradation and amorphization than AlN and SiC ceramics, which are more resistant to the influence of electronic interactions and the consequences caused by them. The higher resistance to the influence of electron losses of incident ions for AlN and SiC ceramics is expressed in the absence of experimental data confirming the presence of latent tracks in these materials during irradiation with heavy ions.

On the other hand, high-temperature irradiation, at high fluences, can lead to an increase in the size of point defects, as well as the formation of dislocation loops and complex defects, which can also affect the strength properties of ceramics. For example, it was shown in [36] that an increase in the irradiation temperature leads to an increase in the defect density at high irradiation doses, as well as to the formation of complex defects, while for samples irradiated at room temperature, the defect density remains virtually unchanged at high irradiation fluences. The formation of complex defects, as well as dislocation loops, can lead to a disordered structure and thereby reduce the resistance to the formation of microcracks under mechanical action.

Another important characteristic of ceramics, which affects the definition of their application as well as the efficiency of their use as structural materials, is their thermal-physical properties. When these ceramics are used as first wall materials or inert matrices, their heat-transfer properties play an important role. The destruction of material as a result of the accumulation of radiation damage can lead to additional barriers to heat transfer, thereby reducing the heat removal from the core or creating local areas of overheating, which can have a negative impact on the performance of the entire reactor unit. In this connection, the investigated samples were tested before and after irradiation for the preservation of thermal conductivity parameters, as well as for the determination of the influence of degradation processes caused by irradiation on heat exchange and heat losses of irradiated ceramics.

**Figure 3:**
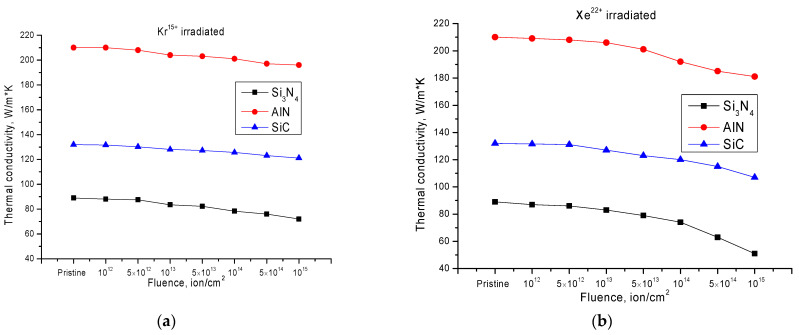
The results of changes in the thermal conductivity coefficient of the investigated ceramics depending on the type of incident ions: (**a**) irradiation with Kr^15+^ ions; (**b**) irradiation with Xe^22+^ ions.

The results of changes in the value of the thermal conductivity coefficient depending on the fluence of the flying ions are shown in Figure 3. The measurements were made in the range of 100 to 800 °C in order to determine the stability of the thermal conductivity coefficient as a function of temperature. Figure 3 shows the data of the average value of the thermal conductivity coefficient.

The general view of changes in the value of the thermal conductivity coefficient for samples irradiated with Kr^15+^ ions indicates that the selected ceramics are sufficiently resistant to changes in thermal physical properties as a result of radiation damage. At small fluences of irradiation 10^12^–10^13^ ions/cm^2^, the change in thermal conductivity is practically not observed, and insignificant fluctuations of thermal conductivity coefficient value can be explained by a measurement error which amounted to no more than 1–2%. This lack of change in thermal conductivity at low irradiation fluences can be explained by the effects of the dynamic annealing of formed point defects during high-temperature irradiation, as well as by the isolation of defect regions formed along the trajectory of the ions in the material. An increase in the irradiation fluence, and hence an increase in the probability of overlapping defect regions, leads to a decrease in the annihilation effect of point defects in the structure due to changes in the value of thermal vibrations of the crystal lattice, which leads to a decrease in thermal conductivity due to changes in the defect concentration in the damaged layer. Additionally, the small changes in the thermal conductivity of irradiated ceramics as compared to data from the literature [27,28] are due to the fact that the samples were irradiated at 1000 K, which contributes to the partial annealing of simple defects, such as vacancies, embedded atoms, or dislocations, which have a strong effect on the thermal conductivity of materials.

At the same time, the greatest changes in the reduction in the thermal conductivity coefficient during irradiation with Kr^15+^ ions are observed for Si_3_N_4_ ceramics, the decrease in thermal conductivity for which at high fluences of irradiation was 15–20% in comparison with the initial values. However, for AlN and SiC the change in thermal conductivity at irradiation fluences of 10^14^–10^15^ ions/cm^2^ was not more than 6–8% in comparison with the initial values. This behavior of the thermal-physical properties for the selected types of ceramics has a correlation with the change in strength properties, which indicates that the deformation effect caused by irradiation also affects the thermal physical properties of the ceramics. However, for Si_3_N_4_ ceramics, the degradation of strength properties is much greater than the change in thermal physical properties, which indicates that the thermal physical properties are greatly affected by electronic ion losses and, consequently, by the change in electronic density in the irradiated layer. This is also evidenced by the results of changes in the thermal conductivity of ceramics under irradiation with Xe^22+^ ions, for which the decrease in thermal physical parameters is more significant than under irradiation with Kr^15+^ ions.

## 4. Conclusions

Analyzing the obtained data, we can conclude that the change in the strength properties of ceramics is significantly affected by nuclear losses of incident ions leading to the displacement of atoms from the lattice nodes, as well as the formation of complex defects and dislocation loops at high fluences of irradiation. Moreover, the greatest changes in the strength properties under irradiation with Kr^15+^ and Xe^22+^ heavy ions were observed for Si_3_N_4_ ceramics, while for AlN and SiC samples, the change in hardness and resistance to softening and degradation was much less evident than for Si_3_N_4_ ceramics. In the case of changes in the thermal-physical properties of ceramics, the most significant changes were observed during irradiation with Xe^22+^ ions, which can be explained by the formation of additional defects in the ceramics structure, scattering which leads to reduced heat transfer and decreased thermal conductivity.

Further prospects for research on these ceramics are related to a detailed study of the effect of high-temperature irradiation on structural properties, including establishing the relationship between changes in strength and thermal-physical parameters with defect formation mechanisms, as well as establishing the concentration of amorphous-like or disordered areas in the structure of the damaged layer using electron microscopy and X-ray analysis.

## Figures and Tables

**Table 1:** Data on the energy losses of the incident ions.

Type of Ceramics	Kr^15+^			Xe^22+^		
	dE/dx_electron_, keV/Micron	dE/dx_nuclear_, keV/Micron	Projected Range, Micron	dE/dx_electron_, keV/Micron	dE/dx_nuclear_, keV/Micron	Projected Range, Micron
Si_3_N_4_	13,600	29	15.19	21,400	66	16.61
AlN	13,900	30	15.04	21,900	65	16.01
SiC	13,900	30	14.59	21,800	66	15.52

## Data Availability

The data presented in this study are available on request from the corresponding authors.
